# Conteltinib (CT-707) in patients with advanced ALK-positive non-small cell lung cancer: a multicenter, open-label, first-in-human phase 1 study

**DOI:** 10.1186/s12916-022-02646-0

**Published:** 2022-11-23

**Authors:** Puyuan Xing, Qian Zhao, Li Zhang, Hanping Wang, Dingzhi Huang, Pei Hu, Yinghui Sun, Yuankai Shi

**Affiliations:** 1 Department of Medical Oncology, National Cancer Center/National Clinical Research Center for Cancer/Cancer Hospital, Chinese Academy of Medical Sciences & Peking Union Medical College, Beijing Key Laboratory of Clinical Study on Anticancer Molecular Targeted Drugs, No. 17 Panjiayuan Nanli, Chaoyang District, Beijing, 100021 China; 2 Clinical Pharmacology Research Center, Peking Union Medical College Hospital, State Key Laboratory of Complex Severe and Rare Diseases, NMPA Key Laboratory for Clinical Research and Evaluation of Drug, Beijing Key Laboratory of Clinical PK & PD Investigation for Innovative Drugs, Chinese Academy of Medical Sciences & Peking Union Medical College, Beijing, 100730 China; 3 Department of Respiratory and Critical Care Medicine, Peking Union Medical College Hospital, Chinese Academy of Medical Sciences & Peking Union Medical College, Beijing, 100730 China; 4 Department of Thoracic Oncology, Tianjin Medical University Cancer Institute & Hospital, Tianjin, 300060 China; 5 Department of Clinical Medicine, Shouyao Holdings (Beijing) Co., Ltd, Beijing, 100195 China

**Keywords:** Non-small cell lung cancer, Anaplastic lymphoma kinase, Tyrosine kinase inhibitor, Conteltinib, CT-707, Phase 1 study

## Abstract

**Background:**

Conteltinib (CT-707) is a potent second-generation anaplastic lymphoma kinase (ALK) tyrosine kinase inhibitor (TKI) showing promising anti-tumor activities in preclinical studies. This study aimed to assess the safety, pharmacokinetic (PK), and efficacy of conteltinib in patients with ALK-positive non-small cell lung cancer (NSCLC).

**Methods:**

In this multicenter, single-arm, open-label, first-in-human phase 1 study, conteltinib was taken orally at doses of 50 to 800 mg quaque die (QD) in a dose-escalation phase. If the response was observed in a dose cohort of the dose-escalation phase, dose expansion was started. The primary endpoints were maximum tolerated dose (MTD), dose-limiting toxicity (DLT), and adverse events assessed by investigators.

**Results:**

Between April 13, 2016, and February 8, 2020, 64 ALK-positive NSCLC patients were enrolled, including 41 (64.1%) patients with ALK TKI-naïve and 23 (35.9%) patients who received crizotinib previously. In the dose-escalation phase, 26 patients were treated with conteltinib at doses of 50 mg, 100 mg, 200 mg, 300 mg, 450 mg, 600 mg, and 800 mg QD. One DLT event was reported at the dose of 600 mg. MTD was not reached. Overall, 58 (90.6%) patients experienced treatment-related adverse events (TRAEs) and 9 (14.1%) patients had grade ≥ 3 TRAEs. The most common TRAEs were diarrhea (46 [71.9%]), serum creatinine elevated (29 [45.3%]), aspartate aminotransferase elevated (25 [39.1%]), and nausea (24 [37.5%]). Among 39 ALK TKI-naïve patients, the overall response rate (ORR) was 64.1% (25 of 39; 95% confidence interval [CI], 47.2–78.8), median progression-free survival (PFS) was 15.9 months (95% CI, 9.26–23.3), and median duration of response (DoR) was 15.0 months (95% CI, 9.06–25.8). Among 21 patients who received crizotinib previously, the ORR was 33.3% (7 of 21; 95% CI, 14.6–57.0), median PFS was 6.73 months (95% CI, 4.73–8.54), and median DoR was 6.60 months (95% CI, 3.77–13.3).

**Conclusions:**

In this study, conteltinib showed manageable safety profile, favorable PK properties, and anti-tumor activity in advanced ALK-positive NSCLC patients. The recommended phase 2 dose was determined to be 600 mg QD for ALK TKI-naïve patients and 300 mg bis in die (BID) for patients who received crizotinib previously.

**Trial registration:**

ClinicalTrials.gov, NCT02695550.

**Supplementary Information:**

The online version contains supplementary material available at 10.1186/s12916-022-02646-0.

## Background

Genetic alterations, including mutation, gene amplification, or chromosomal rearrangement in the *anaplastic lymphoma kinase* (*ALK*) gene, are detected in 3 to 8% of patients with non-small cell lung cancer (NSCLC) [1, 2]. As a receptor tyrosine kinase, the aberrantly activated *ALK* leads to the expression of a potent oncogenic driver and triggers oncogenic signaling pathways, principally PI3K, JAK/STAT, and RAS/MEK/ERK pathways [3].

Crizotinib was the first US Food and Drug Administration (FDA)-approved ALK tyrosine kinase inhibitor (TKI) and has high efficacy in locally advanced or metastatic NSCLC patients harboring ALK rearrangement, with response rates of approximately 60% across multiple studies and a median progression-free survival (PFS) of 8–10 months [4, 5]. Despite experiencing initial responses, most patients inevitably relapse within 1 year, owing to the development of resistance [6]. The mechanisms were complex and heterogeneous, including on-target mechanisms such as ALK tyrosine kinase domain mutation and ALK fusion gene amplification [7–9] and off-target mechanisms such as activation of bypass signaling pathways [10–12].

Several second-generation ALK TKIs, such as ceritinib, alectinib, brigatinib, ensartinib, and iruplinalkib, and third-generation ALK TKIs, such as lorlatinib, have been developed to overcome crizotinib resistance. These ALK TKIs have achieved clinical benefits in crizotinib-refractory patients, with a median PFS of 6.9–12.9 months [13–18]. Moreover, in ALK TKI-naïve, ALK-positive advanced NSCLC patients, these ALK TKIs are more efficacious, with a median PFS of 18.4–34.8 months [13, 16, 17, 19, 20]. Central nervous system (CNS) metastases are highly prevalent in patients with ALK-positive advanced NSCLC and the most common metastasis types in patients receiving crizotinib treatment [21]. Of note, the next-generation ALK TKIs have better CNS penetration, showing efficacy in controlling brain metastases [13, 17, 22, 23].

Conteltinib (CT-707) is an oral, highly potent and ATP-competitive, second-generation ALK TKI developed by Shouyao Holdings (Beijing) Co., Ltd, Beijing, China. In enzymatic assays, conteltinib is more potent (about 10-fold) than crizotinib against ALK and can inhibit various crizotinib-resistant mutations including L1196M, G1202R, F1174L, G1269S, and R1275Q in ALK kinase domain (Additional file 1: Table S1). In addition, conteltinib also inhibits FAK and Pyk2, although less potent than the inhibition to ALK [24]. In xenograft models of ALK-positive NSCLC, conteltinib showed marked anti-tumor activity both in crizotinib-sensitive and crizotinib-resistant tumors [25]. The preclinical studies suggest that conteltinib may be active in ALK-positive NSCLC patients with ALK TKI-naïve, as well as those who have had disease progression during crizotinib treatment. This was a phase 1 study (NCT02695550) to determine the safety, maximum tolerated dose (MTD), pharmacokinetic (PK), and anti-tumor activity of conteltinib in patients with advanced ALK-positive NSCLC.

## Methods

### Clinical study summary

This was a first-in-human, single-arm, multicenter, open-label, phase 1, dose-escalation, and dose-expansion study (NCT02695550) of conteltinib in patients with ALK-positive NSCLC. The study was conducted in three hospitals in China and in accordance with all applicable regulatory requirements and had institutional review board approval prior to study initiation at participating hospitals. Written informed consents were obtained from all individual participating patients prior to the initiation of the study.

### Study design

In the dose-escalation phase, the dose was escalated sequentially from a low to a high level with a 3 + 3 dose-escalation scheme based on the modified Fibonacci method. The starting dose for conteltinib was 50 mg orally quaque die (QD). Dose escalation proceeded to the next six cohorts of 100 mg, 200 mg, 300 mg, 450 mg, 600 mg, and 800 mg QD. For each dose, there was a PK lead-in phase where a single dose was given 7 days before cycle 1 day 1. Then, each dose was taken continuously in a 28-day cycle. The first cycle was for dose-limiting toxicity (DLT) investigation. When one of three patients experienced a DLT in a dose cohort, three additional patients were enrolled in the same dose cohort to assess the dose level in a total of six patients. All patients received conteltinib until disease progression, unacceptable toxicity, or withdrawal of consent. Patients received follow-up safety assessments for 30 days (±7 days) after the last conteltinib dose.

If the response was observed in a dose cohort of the dose-escalation phase, dose expansion was started. In the dose-expansion phase, each dose was taken continuously in a 28-day cycle. At the end of each cycle, a safety evaluation was conducted. If patients met the criteria to enter the next cycle, treatment with conteltinib was continued for subsequent cycles. All patients received conteltinib until disease progression, unacceptable toxicity, or withdrawal of consent. Patients received follow-up safety assessments for 30 days (±7 days) after the last dose of conteltinib.

### Study population

Patients were eligible for this study if they met the following criteria: adult patients (18–75 years of age) with Eastern Cooperative Oncology Group (ECOG) performance status (PS) ≤ 2; estimated life expectancy ≥ 12 weeks; histologically or cytologically confirmed diagnosis of advanced ALK-positive NSCLC as determined by fluorescence in situ hybridization (FISH), immunohistochemistry (IHC), polymerase chain reaction (PCR), or next-generation sequencing (NGS); must have at least one measurable lesion; no or asymptomatic brain metastases or symptomatic brain metastasis which remained stable for > 4 weeks after treatment; adequate organ function, including bone marrow function (absolute neutrophil count ≥ 1.5×10^9^/L, platelets ≥ 100×10^9^/L, hemoglobin ≥ 90 g/L), liver function (total bilirubin ≤ 1.5× upper limit of normal [ULN], alanine aminotransferase (ALT) or aspartate aminotransferase (AST) ≤ 2.5× ULN [≤ 5.0× ULN for liver metastasis patients]), renal function (creatinine clearance ≥ 60 mL/min), cardiac function (left ventricular ejection fraction [LVEF] ≥ 50%), blood glucose (fasting blood glucose ≤ 200 mg/dL), and electrocardiogram (ECG)-corrected QT interval (QTc) of < 450 ms in males or < 470 ms in females; no major surgery within 6 weeks or radiotherapy/minor surgery within 2 weeks (palliative radiotherapy within 48 h) prior to first dose of conteltinib and that any prior toxicity from anti-tumor therapy had been resolved to at least grade 1 (except hair loss).

Key exclusion criteria included any of the following within 6 months prior to study enrollment: myocardial infarction, severe/unstable angina pectoris, coronary/peripheral artery bypass graft, congestive heart failure and cerebrovascular accident or transient ischemic attack; uncontrolled nausea, vomiting, or diarrhea of grade ≥ 1; peripheral neuropathy of grade ≥ 3; active and clinically significant bacterial, fungal, or viral infection (hepatitis B, hepatitis C, human immunodeficiency virus [HIV], or acquired immunodeficiency syndrome [AIDS]-related illness); receipt of any compound known to be a potent inducer or inhibitor of CYP3A4; history of extensive disseminated or bilateral or known presence of grade 3 or 4 interstitial fibrosis or interstitial lung disease; history of other malignancies except cured basal cell carcinoma of skin and carcinoma in situ of uterine cervix and gastrointestinal dysfunction or gastrointestinal diseases that may significantly affect the absorption of conteltinib.

### Safety assessments

The safety of conteltinib was assessed in safety set (SS) which was based on the National Cancer Institute Common Terminology Criteria for Adverse Events (CTCAE) version 4.03, laboratory tests, vital signs, ECG, and clinical observations. SS included patients who received at least one dose of conteltinib. Adverse events (AEs) were judged to be treatment-related or not by investigators.

DLTs were AEs occurring in the first cycle of treatment (28 days) that were attributed to conteltinib, and the criteria were grade 4 neutropenia lasting ≥ 7 days, febrile neutropenia, grade 3 thrombocytopenia, grade 3 non-hematologic toxicity, and any conteltinib-related toxicity resulting in treatment delay > 2 weeks or discontinuation of conteltinib treatment at the assigned dose level. Besides, grade 3 nausea, vomiting, or diarrhea for ≥ 3 days or grade 4 nausea, vomiting, or diarrhea of any duration was considered as a DLT. The MTD was defined as the dose of conteltinib that would be closest to but not higher than a 33% probability of a DLT.

### PK assessments

PK analysis set (PKAS) was defined as all enrolled patients who received at least one dose of conteltinib and provided at least one measurable post-dose plasma sample. For single-dose PK parameters, plasma samples were taken at pre-dose and 0.5 h, 1 h, 2 h, 3 h, 4 h, 6 h, 8 h, 12 h, 24 h, 48 h, 72 h, 96 h, 120 h, 144 h, and 168 h post-dose in PK lead-in phase of dose-escalation phase, and plasma samples were also taken at pre-dose and 0.5 h, 1 h, 2 h, 3 h, 4 h, 6 h, 8 h, 12 h, and 24 h post-dose in cycle 1 day 1 of dose-expansion phase. During continuous dosing, serial plasma samples were collected at pre-dose in cycle 1 day 15, pre-dose in cycle 1 day 22, pre-dose and 0.5 h, 1 h, 2 h, 3 h, 4 h, 6 h, 8 h, 12 h, and 24 h post-dose in cycle 1 day 28 for all dose cohorts. The following PK parameters were assessed: area under the concentration-time curve from the time of dosing extrapolated to infinity, based on the last observed concentration (AUC_INF_obs_); area under the concentration-time curve from the time of dosing to the time of last observation (AUC_last_); maximum concentration (*C*_max_); time to maximum concentration (*T*_max_); elimination half-life (*T*_1/2_); and mean residence time from the time of dosing to the time of the last measurable concentration (MRT_last_).

### Efficacy assessments

Patients underwent baseline tumor imaging examination, including computed tomography (CT) or magnetic resonance imaging (MRI) of the brain, chest, abdomen, and pelvis, as clinically indicated. Efficacy imaging examination was performed at the end of cycle 1 and thereafter every 8 weeks till cycle 29 and every 12 weeks then till disease progression which were judged by investigators according to the Response Evaluation Criteria in Solid Tumors (RECIST) version 1.1. Full analysis set (FAS) included patients who received at least one dose of conteltinib and completed at least one post-baseline measurement for efficacy analysis.

### Study endpoints

The primary endpoints for this study were to determine the MTD, as well as DLT and AE including TRAE and serious adverse event (SAE) assessed by investigators.

Secondary endpoints were PK parameters such as *C*_max_, AUC_INF_obs_, AUC_last_, *T*_max_, *T*_1/2_, and MRT_last_, as well as preliminary anti-tumor activity of conteltinib including overall response rate (ORR), disease control rate (DCR), duration of response (DoR), and PFS assessed by investigators.

ORR was defined as the proportion of patients who had the best overall response, including complete response (CR) or partial response (PR). PFS was calculated from the date of the first dose of conteltinib to the date of documented disease progression or death (whichever occurred first). DCR was defined as the proportion of patients who had the best overall response, including CR, PR, or stable disease (SD), with a duration of at least 12 weeks. DoR was calculated from the date when first documented CR or PR (subsequently confirmed) was observed to the date of first documented disease progression or death (whichever occurred first). PFS was defined as the time from the date of the first dose of conteltinib until progressive disease (PD) or death.

### Statistical analyses

There was no formal hypothesis testing in both dose-escalation phase and dose-expansion phase of this study.

Descriptive statistics were carried out to summarize patients’ baseline characteristics and safety. The response rates with exact binomial 95% confidence intervals (CIs) were calculated using the Clopper-Pearson method. All time-to-event data such as PFS, DoR, and their corresponding 95% CIs were estimated by the Kaplan-Meier method. PK parameter analysis was calculated with Phoenix WinNonlin version 8.0. All other statistical analyses were conducted using SAS version 9.4.

## Results

### Patient baseline characteristics

**Fig. 1:**
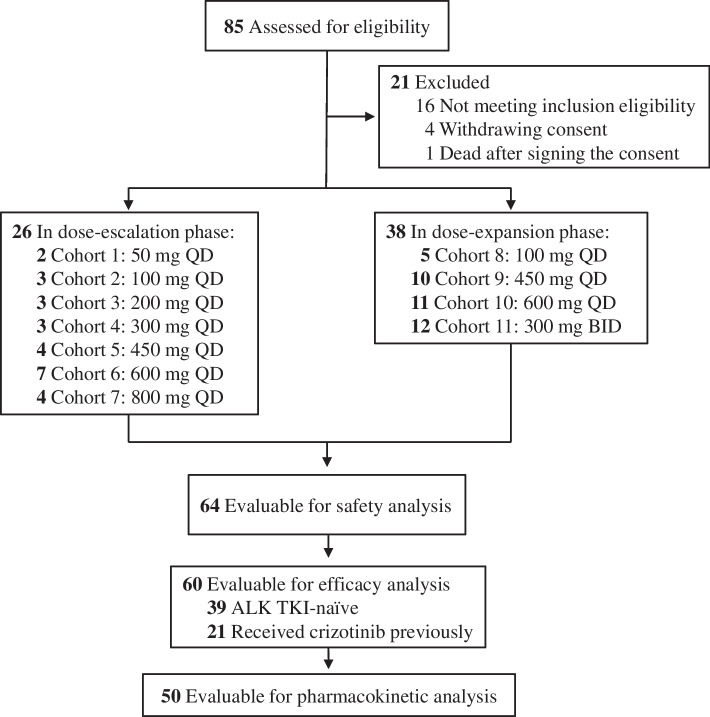
Study flowchart. ALK, anaplastic lymphoma kinase; TKI, tyrosine kinase inhibitor; QD, quaque die; BID, bis in die

Between April 13, 2016, and February 8, 2020, 85 patients with ALK-positive advanced NSCLC were screened, while 21 of which were excluded including 16 patients who did not meet inclusion eligibility, 4 patients who withdrew consent, and 1 patient who died after signing the consent. A total of 64 patients were enrolled (26 patients in the dose-escalation phase and 38 patients in the dose-expansion phase) across 3 hospitals in China and most of them accepted at least one dose of conteltinib treatment (Fig. 1). Patient baseline characteristics are summarized in Table 1. The *ALK*-testing methods included IHC (30/64, 46.9%), FISH (24/64, 37.5%), PCR (9/64, 14.0%), and NGS (1/64, 1.6%); the mean age of patients was 51.3 years (range 31–70); and 96.9% (62 of 64) of patients were with adenocarcinoma. For prior chemotherapy, 20.3% (13 of 64) of patients had never received, 46.9% (30 of 64) had received one chemotherapy regimen, 23.4% (15 of 64) had received two chemotherapy regimens, and 9.4% (6 of 6.4) had received three or more prior chemotherapy regimens. For prior ALK TKI treatment, 35.9% (23 of 64) of patients received crizotinib previously, and 64.1% (41 of 64) of patients were ALK TKI-naïve. At the data cut-off date of February 28, 2022, 95.3% (61 of 64) of patients had discontinued treatment because of disease progression (*n*=57; 89.1%) and were no longer willing to participate or other reasons (*n*=4; 6.3%); 3 patients were still receiving treatment.

**Table 1:** Patient baseline characteristics

Characteristic	Total (***n***=64)	Dose-escalation phase (***n***=26)	Dose-expansion phase (***n***=38)
Age, years			
Mean (SD)	51.3 (9.1)	51.4 (8.5)	51.2 (9.6)
Sex, *n* (%)			
Male	32 (50.0)	15 (57.7)	17 (44.7)
Female	32 (50.0)	11 (42.3)	21 (55.3)
Tumor histology, *n* (%)			
Adenocarcinoma	62 (96.9)	26 (100.0)	36 (94.7)
Others	2 (3.1)	0 (0.0)	2 (5.3)
*ALK-*testing method			
IHC	30 (46.9)	10 (38.5)	20 (52.6)
FISH	24 (37.5)	12 (46.2)	12 (31.6)
PCR	9 (14.0)	3 (11.5)	6 (15.8)
NGS	1 (1.6)	1 (3.8)	0 (0.0)
ECOG PS, *n* (%)			
0	48 (75.0)	19 (73.1)	29 (76.3)
1	16 (25.0)	7 (26.9)	9 (23.7)
Smoking status, *n* (%)			
Presence	19 (29.7)	12 (46.2)	7 (18.4)
Absence	45 (70.3)	14 (53.8)	31 (81.6)
Prior chemotherapy, *n* (%)			
None	13 (20.3)	2 (7.7)	11 (28.9)
One regimen	30 (46.9)	13 (50.0)	17 (44.7)
Two regimens	15 (23.4)	7 (26.9)	8 (21.1)
Three or more regimens	6 (9.4)	4 (15.4)	2 (5.3)
Prior ALK TKIs, *n* (%)			
ALK TKI-naïve	41 (64.1)	14 (53.8)	27 (71.1)
Received crizotinib previously	23 (35.9)	12 (46.2)	11 (28.9)

*Abbreviations*: *ALK* anaplastic lymphoma kinase, *ECOG* Eastern Cooperative Oncology Group, *PS* performance status, *SD* standard deviation, *IHC* immunohistochemistry, *FISH* fluorescence in situ hybridization, *PCR* polymerase chain reaction, *NGS* next-generation sequencing, *TKI* tyrosine kinase inhibitor

### Safety

Twenty-six patients were enrolled in the dose-escalation phase, 2 of 26 patients withdrew consents and 24 of 26 patients received conteltinib treatment: 50 mg QD (*n*=2), 100 mg QD (*n*=3), 200 mg (*n*=3), 300 mg QD (*n*=3), 450 mg QD (*n*=3), 600 mg QD (*n*=7), and 800 mg QD (*n*=3), respectively (Fig. 1). One DLT event (grade 3 serum creatinine elevated) occurred during cycle 1 in a patient receiving 600 mg QD, and the DLT resolved after dose reduction of conteltinib. No DLTs were observed in the 800 mg QD cohort; thus, the MTD was not reached in this study. In the dose-expansion phase, 38 patients were enrolled with 1 patient withdrawing consent, the remaining 37 patients were assigned into 100 mg QD (*n*=5), 450 mg QD (*n*=10), 600 mg QD (*n*=11), or 300 mg bis in die (BID) (*n*=11), and safety was assessed at those doses. No DLTs were observed in the dose-expansion phase.

All the 64 patients entered in SS. TRAEs occurred in 58 (90.6%) patients. Most of the TRAEs were grade 1–2 and 9 (14.1%) patients had grade ≥3 TRAEs. The most common TRAEs (≥10%) were diarrhea (71.9%), serum creatinine elevated (45.3%), AST elevated (39.1%), nausea (37.5%), vomiting (35.9%), ALT elevated (34.4%), γ-glutamyl transpeptidase (γ-GGT) elevated (32.8%), hyperuricemia (31.25%), weight loss (28.1%), abdominal pain (26.6%), hypertriglyceridemia (23.4%), proteinuria (21.9%), appetite decreased (18.75%), hypoalbuminemia (17.2%), abdominal pain upper (17.2%), hyperglycemia (15.6%), hypercholesterolemia (12.5%), and conjugated bilirubin elevated (10.9%). The most common grade ≥ 3 TRAEs were γ-GGT elevated (7.8%), diarrhea (3.1%), serum creatinine elevated (1.6%), and conjugated bilirubin elevated (1.6%) (Table 2). All of which were reversible on discontinuation of conteltinib treatment.

**Table 2:** Most common TRAEs (≥10%) of conteltinib (CT-707) in SS

TRAEs ***n*** (%)	Total (***n***=64)			50 mg QD (***n***=2)		100 mg QD (***n***=8)		200 mg QD (***n***=3)		300 mg QD (***n***=3)		450 mg QD (***n***=14)		600 mg QD (***n***=18)		800 mg QD (***n***=4)		300 mg BID (***n***=12)	
	All grade	Grade 1–2	Grade ≥ 3	Grade 1–2	Grade ≥ 3	Grade 1–2	Grade ≥ 3	Grade 1–2	Grade ≥ 3	Grade 1–2	Grade ≥ 3	Grade 1–2	Grade ≥ 3	Grade 1–2	Grade ≥ 3	Grade 1–2	Grade ≥ 3	Grade 1–2	Grade ≥ 3
Patients with any TRAE	58 (90.6)	58 (90.6)	9 (14.1)	1 (50.0)	0 (0.0)	6 (75.0)	0 (0.0)	3 (100)	0 (0.0)	3 (100)	0 (0.0)	14 (100)	3 (21.4)	18 (100)	5 (27.8)	3 (75.0)	0 (0.0)	10 (83.3)	1 (8.3)
Diarrhea	46 (71.9)	44 (68.8)	2 (3.1)	1 (50.0)	0 (0.0)	0 (0.0)	0 (0.0)	0 (0.0)	0 (0.0)	3 (100)	0 (0.0)	13 (92.9)	0 (0.0)	16 (88.9)	2 (11.1)	3 (75.0)	0 (0.0)	8 (66.7)	0 (0.0)
Serum creatinine elevated	29 (45.3)	28 (43.8)	1 (1.6)	0 (0.0)	0 (0.0)	2 (25.0)	0 (0.0)	0 (0.0)	0 (0.0)	1 (33.3)	0 (0.0)	10 (71.4)	0 (0.0)	8 (44.4)	1 (5.6)	2 (50.0)	0 (0.0)	5 (41.7)	0 (0.0)
AST elevated	25 (39.1)	25 (39.1)	0 (0.0)	0 (0.0)	0 (0.0)	2 (25.0)	0 (0.0)	1 (33.3)	0 (0.0)	1 (33.3)	0 (0.0)	9 (64.3)	0 (0.0)	8 (44.4)	0 (0.0)	1 (25.0)	0 (0.0)	3 (25.0)	0 (0.0)
Nausea	24 (37.5)	24 (37.5)	0 (0.0)	0 (0.0)	0 (0.0)	3 (37.5)	0 (0.0)	0 (0.0)	0 (0.0)	1 (33.3)	0 (0.0)	6 (42.9)	0 (0.0)	9 (50.0)	0 (0.0)	1 (25.0)	0 (0.0)	4 (33.3)	0 (0.0)
Vomiting	23 (35.9)	23 (35.9)	0 (0.0)	0 (0.0)	0 (0.0)	1 (12.5)	0 (0.0)	0 (0.0)	0 (0.0)	1 (33.3)	0 (0.0)	6 (42.9)	0 (0.0)	11 (61.1)	0 (0.0)	2 (50.0)	0 (0.0)	2 (16.7)	0 (0.0)
ALT elevated	22 (34.4)	22 (34.4)	0 (0.0)	0 (0.0)	0 (0.0)	1 (12.5)	0 (0.0)	0 (0.0)	0 (0.0)	1 (100)	0 (0.0)	10 (71.4)	0 (0.0)	7 (38.9)	0 (0.0)	0 (0.0)	0 (0.0)	3 (25.0)	0 (0.0)
γ-GGT elevated	21 (32.8)	16 (25.0)	5 (7.8)	0 (0.0)	0 (0.0)	0 (0.0)	0 (0.0)	0 (0.0)	0 (0.0)	0 (0.0)	0 (0.0)	3 (21.4)	3 (21.4)	8 (44.4)	1 (5.6)	2 (50.0)	0 (0.0)	3 (25.0)	1 (8.3)
Hyperuricemia	20 (31.3)	20 (31.3)	0 (0.0)	0 (0.0)	0 (0.0)	1 (12.5)	0 (0.0)	1 (33.3)	0 (0.0)	0 (0.0)	0 (0.0)	3 (21.4)	0 (0.0)	10 (55.6)	0 (0.0)	3 (75.0)	0 (0.0)	2 (16.7)	0 (0.0)
Weight loss	18 (28.1)	18 (28.1)	0 (0.0)	0 (0.0)	0 (0.0)	0 (0.0)	0 (0.0)	0 (0.0)	0 (0.0)	3 (100)	0 (0.0)	3 (21.4)	0 (0.0)	8 (44.4)	0 (0.0)	2 (50.0)	0 (0.0)	2 (16.7)	0 (0.0)
Abdominal pain	17 (26.6)	17 (26.6)	0 (0.0)	0 (0.0)	0 (0.0)	0 (0.0)	0 (0.0)	0 (0.0)	0 (0.0)	0 (0.0)	0 (0.0)	5 (35.7)	0 (0.0)	7 (38.9)	0 (0.0)	3 (75.0)	0 (0.0)	2 (16.7)	0 (0.0)
Hypertriglyceridemia	15 (23.4)	15 (23.4)	0 (0.0)	0 (0.0)	0 (0.0)	1 (12.5)	0 (0.0)	0 (0.0)	0 (0.0)	1 (33.3)	0 (0.0)	2 (14.3)	0 (0.0)	8 (44.4)	0 (0.0)	2 (50.0)	0 (0.0)	1 (8.3)	0 (0.0)
Proteinuria	14 (21.9)	14 (21.9)	0 (0.0)	0 (0.0)	0 (0.0)	0 (0.0)	0 (0.0)	0 (0.0)	0 (0.0)	0 (0.0)	0 (0.0)	1 (7.1)	0 (0.0)	8 (44.4)	0 (0.0)	2 (50.0)	0 (0.0)	3 (25.0)	0 (0.0)
Appetite decreased	12 (18.8)	12 (18.8)	0 (0.0)	0 (0.0)	0 (0.0)	0 (0.0)	0 (0.0)	0 (0.0)	0 (0.0)	0 (0.0)	0 (0.0)	2 (14.3)	0 (0.0)	7 (38.9)	0 (0.0)	1 (25.0)	0 (0.0)	2 (16.7)	0 (0.0)
Hypoalbuminemia	11 (17.2)	11 (17.2)	0 (0.0)	0 (0.0)	0 (0.0)	0 (0.0)	0 (0.0)	0 (0.0)	0 (0.0)	1 (33.3)	0 (0.0)	2 (14.3)	0 (0.0)	3 (16.7)	0 (0.0)	1 (25.0)	0 (0.0)	4 (33.3)	0 (0.0)
Abdominal pain upper	11 (17.2)	11 (17.2)	0 (0.0)	0 (0.0)	0 (0.0)	1 (12.5)	0 (0.0)	0 (0.0)	0 (0.0)	0 (0.0)	0 (0.0)	5 (35.7)	0 (0.0)	2 (11.1)	0 (0.0)	0 (0.0)	0 (0.0)	3 (25.0)	0 (0.0)
Hyperglycemia	10 (15.6)	10 (15.6)	0 (0.0)	0 (0.0)	0 (0.0)	1 (12.5)	0 (0.0)	0 (0.0)	0 (0.0)	1 (33.3)	0 (0.0)	1 (7.1)	0 (0.0)	6 (33.3)	0 (0.0)	1 (25.0)	0 (0.0)	0 (0.0)	0 (0.0)
Hypercholesterolemia	8 (12.5)	8 (12.5)	0 (0.0)	0 (0.0)	0 (0.0)	1 (12.5)	0 (0.0)	0 (0.0)	0 (0.0)	1 (33.3)	0 (0.0)	1 (7.1)	0 (0.0)	4 (22.2)	0 (0.0)	1 (25.0)	0 (0.0)	0 (0.0)	0 (0.0)
Conjugated bilirubin elevated	7 (10.9)	6 (9.4)	1 (1.6)	0 (0.0)	0 (0.0)	1 (12.5)	0 (0.0)	0 (0.0)	0 (0.0)	0 (0.0)	0 (0.0)	2 (14.3)	0 (0.0)	2 (11.1)	0 (0.0)	0 (0.0)	0 (0.0)	1 (8.3)	1 (8.3)

*Abbreviations*: *TRAE* treatment-related adverse event, *AST* aspartate aminotransferase, *ALT* alanine aminotransferase, *γ-GGT* γ-glutamyl transpeptidase, *SS* safety set, *QD* quaque die, *BID* bis in die

In this study, TRAEs including serum creatinine elevated and impaired liver function led to conteltinib interruption in 4 patients (6.3%); serum creatinine elevated, neutropenia, and impaired liver function led to conteltinib dose reduction in 3 patients (4.7%); and impaired liver function led to permanent discontinuation of conteltinib in 2 patients (3.1%). No patients died during the treatment of conteltinib.

### PK

Fifty patients were included in PKAS, among which 25 patients were in the dose-escalation phase and 25 patients were in the dose-expansion phase.

**Fig. 2:**
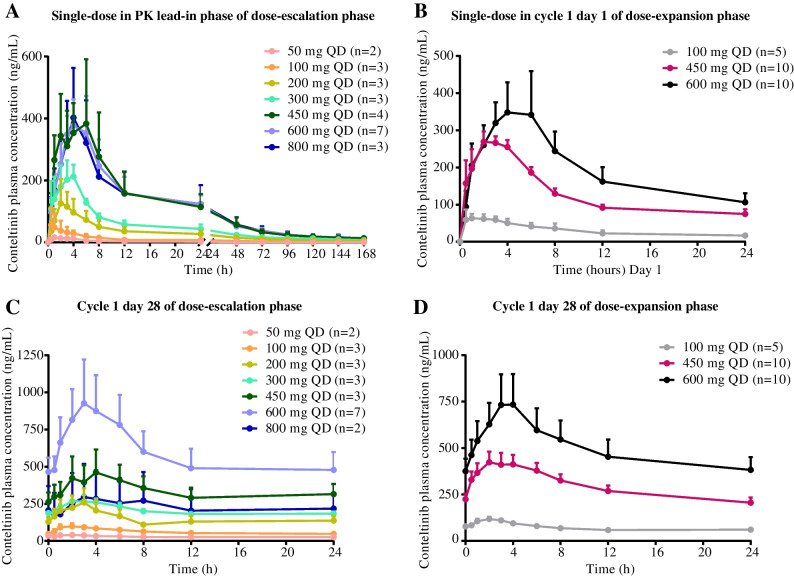
Mean plasma concentration-time profiles of conteltinib (CT-707). **A** Plasma concentration-time curve of a single dose of conteltinib PK assessed in the PK lead-in phase of the dose-escalation phase at pre-dose and 0.5 h, 1 h, 2 h, 3 h, 4 h, 6 h, 8 h, 12 h, 24 h, 48 h, 72 h, 96 h, 120 h, 144 h, and 168 h post-dose. **B** Plasma concentration-time curve of single-dose conteltinib PK assessed in cycle 1 day 1 of dose-expansion phase at pre-dose and 0.5 h, 1 h, 2 h, 3 h, 4 h, 6 h, 8 h, 12 h, and 24 h post-dose. **C** Plasma concentration-time curve of conteltinib PK assessed in cycle 1 day 28 of dose-escalation phase at pre-dose and 0.5 h, 1 h, 2 h, 3 h, 4 h, 6 h, 8 h, 12 h, 24 h post-dose. **D** Plasma concentration-time curve of conteltinib PK assessed in cycle 1 day 28 of dose-expansion phase at pre-dose and 0.5 h, 1 h, 2 h, 3 h, 4 h, 6 h, 8 h, 12 h, 24 h post-dose. Data are mean (standard error of the mean). PK, pharmacokinetic; QD, quaque die; ALK, anaplastic lymphoma kinase; NSCLC, non-small cell lung cancer

For single-dose administration, the mean *T*_max_ for conteltinib ranged from 2.0 to 4.6 h across different dose cohorts of conteltinib, and the mean *T*_1/2_ ranged from 16.7 to 90.2 h. Meanwhile, the area under the concentration-time curve (AUC) and *C*_max_ of conteltinib increased in a manner beyond the dose proportion over the dose range of 50–600 mg. However, *C*_max_, AUC_last_, and AUC_INF_obs_ did not show significant change with dose escalated from 600 to 800 mg, suggesting that the exposure of conteltinib in patients seemed to be saturated in dosage of 600 to 800 mg (Fig. 2A, B).

For multiple-dose administration, the mean *T*_max_ for conteltinib ranged from 1.5 to 4.3 h across different dose cohorts, and the mean *T*_1/2_ ranged from 26.3 to 352.2 h. As for conteltinib exposure, *C*_max_, AUC_last_, and AUC_INF_obs_ increased after repeated dosing in the dose range of 50 to 600 mg, implying the accumulation of conteltinib in patients after repeated oral intake (Fig. 2C, D). The detailed PK parameters of conteltinib in each dose cohort are summarized in Table 3.

**Table 3:** PK parameters of conteltinib (CT-707) in different dose cohorts

PK parameter	AUC_**INF_obs**_ (ng·h/mL)		AUC_**last**_ (ng·h/mL)		***C***_**max**_ (ng/mL)		***T***_**1/2**_ (h)		***T***_**max**_ (h)		MRT_**last**_ (h)	
**Single dose in PK lead-in phase of dose-escalation phase**^a^	Mean	SD	Mean	SD	Mean	SD	Mean	SD	Mean	SD	Mean	SD
50 mg (*n*=2)	205.7	0.6	125.3	15.2	16.3	2.3	22.5	5.9	2.0	1.4	8.1	0.1
100 mg (*n*=3)	1047.4	981.3	732.0	797.6	69.0	85.8	90.2	63.2	2.8	2.0	30.3	15.8
200 mg (*n*=3)	3108.0	1890.6	2553.9	1616.5	139.6	123.6	83.5	6.3	2.7	0.6	47.4	4.4
300 mg (*n*=3)	5109.5	1605.6	4335.8	1818.5	246.3	90.1	78.5	38.5	2.5	1.8	46.2	3.6
450 mg (*n*=4)	11475.2	8250.4	9875.0	7169.1	544.3	385.3	78.7	10.3	3.3	2.2	40.5	1.4
600 mg (*n*=7)	11150.6	6793.3	9644.2	6243.8	452.1	284.6	78.7	13.5	3.9	1.7	43.3	3.7
800 mg (*n*=3)	11164.6	7787.7	9823.2	7130.1	421.3	259.6	77.2	9.3	3.7	0.6	42.0	4.0
**Single-dose in cycle 1 day 1 of dose-expansion phase**^b^	Mean	SD	Mean	SD	Mean	SD	Mean	SD	Mean	SD	Mean	SD
100 mg (*n*=5)	1309.9	552.3	736.7	423.4	85.5	51.0	26.7	16.7	2.7	1.5	8.9	0.9
450 mg (*n*=10)	6339.7	4927.2	3055.1	849.2	327.8	122.0	23.4	19.3	2.3	1.0	9.3	1.2
600 mg (*n*=10)	7153.8	4940.6	4608.9	3267.3	472.4	345.8	16.7	4.0	4.6	2.3	9.8	1.4
**Cycle 1 day 28 of dose-escalation phase**^c^	Mean	SD	Mean	SD	Mean	SD	Mean	SD	Mean	SD	Mean	SD
50 mg (*n*=2)	16704.7	21404.0	683.0	205.5	42.7	4.1	352.2	446.0	1.5	0.7	11.1	0.5
100 mg (*n*=3)	6110.5	5079.7	1479.9	194.0	105.6	29.9	61.1	55.7	2.7	0.6	10.6	0.1
200 mg (*n*=3)	12399.1	8484.5	3573.7	1587.8	259.3	164.3	42.2	20.2	3.0	0.0	11.2	1.1
300 mg (*n*=3)	19006.6	11836.7	4859.1	399.7	282.3	55.9	50.9	36.8	3.0	1.0	11.3	0.7
450 mg (*n*=3)	34382.8	28835.3	8046.3	3449.6	474.0	250.9	48.6	38.2	4.3	1.5	11.6	0.6
600 mg (*n*=7)	53825.9	63563.9	14023.9	8628.0	984.7	717.0	45.0	32.1	2.5	1.4	10.8	0.5
800 mg (*n*=2)	27223.0	33065.7	5498.7	5665.9	304.9	302.8	52.7	30.7	3.5	0.7	11.6	0.1
**Cycle 1 day 28 of dose-expansion phase**^d^	Mean	SD	Mean	SD	Mean	SD	Mean	SD	Mean	SD	Mean	SD
100 mg (*n*=5)	6460.4	2881.3	1709.5	289.1	123.4	23.9	53.2	32.4	2.0	0.7	10.7	0.5
450 mg (*n*=10)	15683.9	9391.2	7040.6	2456.1	491.0	176.8	26.3	11.0	3.0	1.4	10.5	0.6
600 mg (*n*=10)	32508.7	20202.8	11908.9	7300.7	772.1	532.9	37.1	19.4	3.3	1.2	10.9	0.6

*Abbreviations*: *PK* pharmacokinetic, *AUC* area under the concentration-time curve, *AUC*_*INF_obs*_ area under the concentration-time curve from the time of dosing extrapolated to infinity, based on the last observed concentration, *AUC*_*last*_ area under the concentration-time curve from the time of dosing to the time of the last observation, *C*_*max*_ maximum concentration, *T*_*max*_ time to maximum concentration, *T*_*1/2*_ elimination half-life, *MRT*_*last*_ mean residence time from the time of dosing to the time of the last measurable concentration, *SD* standard deviation, *QD* quaque die, *BID* bis in die

^a^Single-dose of conteltinib PK assessed in the PK lead-in phase of the dose-escalation phase at pre-dose and 0.5 h, 1 h, 2 h, 3 h, 4 h, 6 h, 8 h, 12 h, 24 h, 48 h, 72 h, 96 h, 120 h, 144 h, and 168 h post-dose

^b^Single-dose of conteltinib PK assessed in cycle 1 day 1 of the dose-expansion phase at pre-dose and 0.5 h, 1 h, 2 h, 3 h, 4 h, 6 h, 8 h, 12 h, and 24 h post-dose

^c^Conteltinib PK assessed in cycle 1 day 28 of the dose-escalation phase at pre-dose and 0.5 h, 1 h, 2 h, 3 h, 4 h, 6 h, 8 h, 12 h, and 24 h post-dose

^d^Conteltinib PK assessed in cycle 1 day 28 of the dose-expansion phase at pre-dose and 0.5 h, 1 h, 2 h, 3 h, 4 h, 6 h, 8 h, 12 h, and 24 h post-dose

### Efficacy

**Fig. 3:**
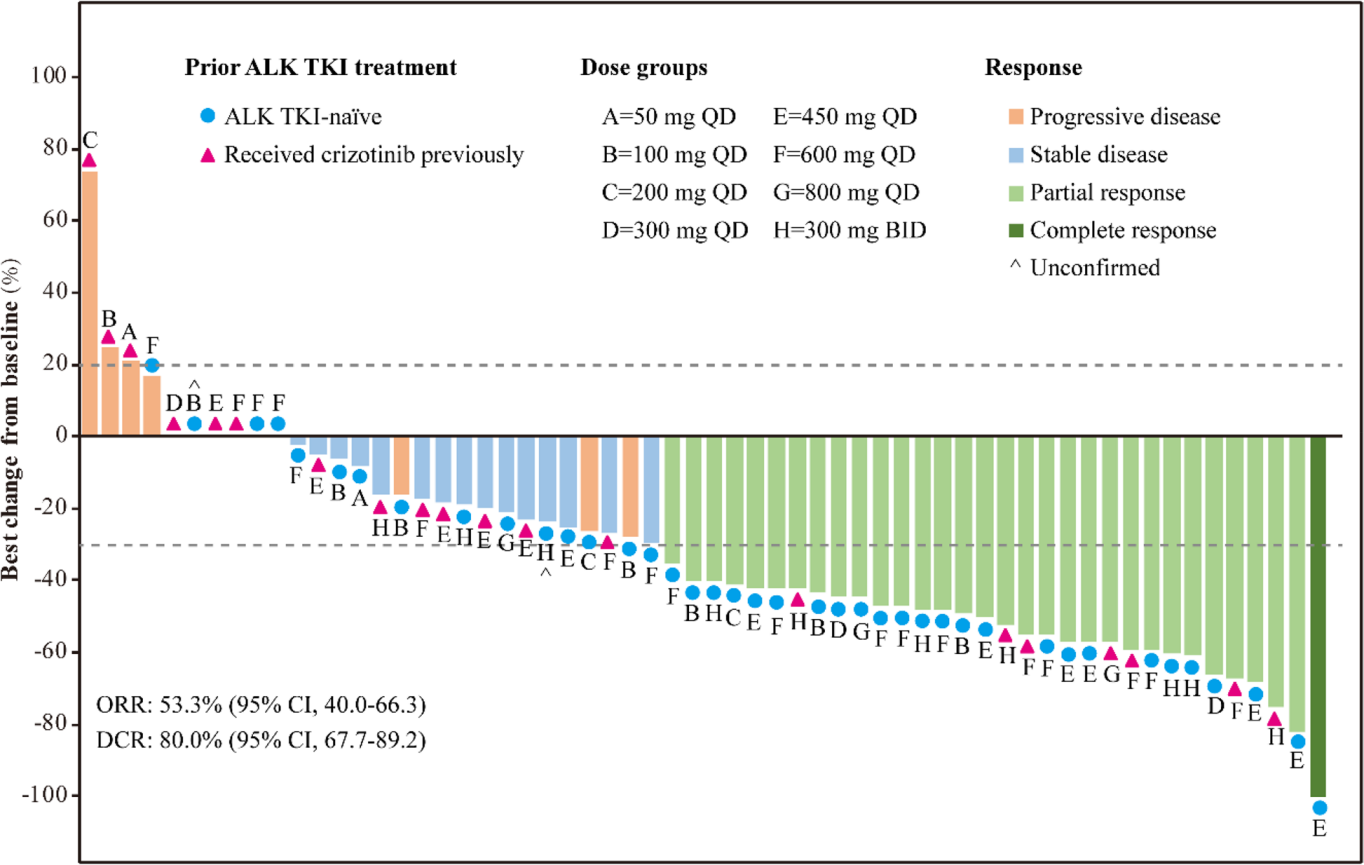
Waterfall plot of conteltinib (CT-707) in patients with ALK-positive NSCLC. Best percentage change from baseline in the sum of target lesions is presented for the ALK-positive evaluable patients (*n*=60). The dashed line at −30% indicates the threshold for PR and that at 20% for PD according to the Response Evaluation Criteria in Solid Tumors version 1.1. One patient achieved CR (dark green), 31 patients achieved PR (green), 18 patients attained SD (blue), and 10 patients experienced PD (pink). **A**–**H** represent patients who received conteltinib at the dose of 50 mg QD, 100 mg QD, 200 mg QD, 300 mg QD, 450 mg QD, 600 mg QD, 800 mg QD, and 300 mg BID, respectively; blue circles refer to patients who are ALK TKI-naïve, and red triangles are patients who received crizotinib previously (note: ^, two patients had unconfirmed response) ALK, anaplastic lymphoma kinase; TKI, tyrosine kinase inhibitor; QD, quaque die; BID, bis in die; NSCLC, non-small cell lung cancer; CR, complete response; PR, partial response; SD, stable disease; PD, progressive disease

Sixty patients with ALK-positive NSCLC who received conteltinib were assessable for efficacy due to 3 patients withdrawing the consent (1 patient of 450 mg QD cohort in dose-escalation phase, 1 patient of 800 mg QD cohort in dose-escalation phase, and 1 patient of 300 mg BID cohort in dose-expansion phase) and 1 patient of 100 mg QD cohort in dose-escalation phase experiencing short dosing time. Among the 60 patients in FAS, at least for 28 days, 1 (1.7%) patient achieved CR, 31 (51.7%) patients achieved PR, 16 (26.7%) patients attained SD, 10 (16.7%) patients experienced PD, and 2 (3.3%) patients were not evaluable (NE). Across all patients, tumor size changes relative to baseline ranged from −100 to 74%, tumor regression was observed in 83.3% (50/60) of the patients (Fig. 3).

Overall, the ORR was 53.3% (95% CI, 40.0–66.3) and DCR was 80.0% (95% CI, 67.7–89.2) in the 60 patients. For patients in four dose-expansion cohorts (100mg QD, 450mg QD, 600 mg QD, and 300 mg BID), the ORR were 42.9% (95% CI, 9.9–81.6), 53.8% (95% CI, 25.1–80.8), 55.6% (95% CI, 30.8–78.5), and 54.5% (95% CI, 23.4–83.3), and DCR were 57.1% (95% CI, 18.4–90.1), 92.3% (95% CI, 64.0–99.8), 83.3% (95% CI, 58.6–96.4), 81.8% (95% CI, 48.2–97.7), respectively (Table 4).

**Table 4:** Efficacy of conteltinib (CT-707) in patients with ALK-positive NSCLC

Response	Total	50 mg QD	100 mg QD	200 mg QD	300 mg QD	450 mg QD	600 mg QD	800 mg QD	300 mg BID
**All patients (*n*)**	60	2	7	3	3	13	18	3	11
ORR (95% CI)	53.3% (40.0–66.3)	50.0% (1.3–98.7)	42.9% (9.9–81.6)	33.3% (0.8–90.6)	66.7% (9.4–99.2)	53.8% (25.1–80.8)	55.6% (30.8–78.5)	66.7% (9.4–99.2)	54.5% (23.4–83.3)
DCR (95% CI)	80.0% (67.7–89.2)	50.0% (1.3–98.7)	57.1% (18.4–90.1)	33.3% (0.8–90.6)	100.0% (29.2–100.0)	92.3% (64.0–99.8)	83.3% (58.6–96.4)	100.0% (29.2–100.0)	81.8% (48.2–97.7)
DoR (months)									
Median (95% CI)	13.3 (6.60–22.2)	NE	11.1 (5.61–15.0)	5.51 (NE–NE)	17.4 (9.06–25.8)	NE (3.74–NE)	15.6 (1.80–22.2)	17.3 (6.60–28.0)	5.58 (1.80–10.6)
PFS (months)									
Median (95% CI)	9.26 (6.73–15.7)	NE (1.21–NE)	8.57 (0.95–15.9)	3.25 (1.34–8.54)	10.2 (4.92–28.8)	26.9 (4.63–NE)	13.2 (4.69–23.0)	10.3 (7.72–29.2)	7.68 (3.05–17.8)
Follow-up time (months)									
Median (95% CI)	9.75 (6.63–12.5)	7.75 (2.98–NE)	9.52 (1.87–17.1)	4.79 (2.33–NE)	11.9 (6.63–NE)	9.82 (4.13–28.7)	14.2 (6.50–24.9)	12.1 (9.33–NE)	6.17 (2.82–18.9)
**ALK TKI-naïve (*n*)**	39	1	6	2	2	8	12	2	6
ORR (95% CI)	64.1% (47.2–78.8)	100.0% (2.5–100.0)	50.0% (11.8–88.2)	50.0% (1.3–98.7)	100.0% (15.8–100.0)	87.5% (47.3–99.7)	58.3% (27.7–84.8)	50.0% (1.3–98.7)	50.0% (11.8–88.2)
DCR (95% CI)	82.1% (66.5–92.5)	100.0% (2.5–100.0)	66.7% (22.3–95.7)	50.0 (1.3–98.7)	100.0% (15.8–100.0)	100.0% (63.1–100.0)	75.0% (42.8–94.5)	100.0% (15.8–100.0)	83.3% (35.9–99.6)
DoR (months)									
Median (95% CI)	15.0 (9.06–25.8)	NE	11.1 (5.61–15.0)	5.51 (NE–NE)	17.4 (9.06–25.8)	NE (3.74–NE)	16.5 (1.80–32.8)	28.0 (NE–NE)	10.6 (1.80–10.6)
PFS (months)									
Median (95% CI)	15.9 (9.26–23.3)	NE	11.3 (2.75–15.9)	5.89 (3.25–8.54)	19.5 (10.2–28.8)	NE (4.63–NE)	19.3 (1.05–32.4)	19.8 (10.3–29.2)	17.8 (3.05–19.6)
Follow-up time (months)									
Median (95% CI)	12.2 (6.17–18.9)	12.5 (NE–NE)	12.7 (3.74–NE)	7.22 (4.79–NE)	21.0 (11.9–NE)	17.1 (2.75–52.5)	20.8 (1.60–33.6)	21.2 (12.1–NE)	6.01 (2.49–NE)
**Received crizotinib previously (*n*)**	21	1	1	1	1	5	6	1	5
ORR (95% CI)	33.3% (14.6–57.0)	0.0% (0.0–97.5)	0.0% (0.0–97.5)	0.0% (0.0–97.5)	0.0% (0.0–97.5)	0.0% (0.0–52.2)	50.0% (11.8–88.2)	100.0% (2.5–100.0)	60.0% (14.7–94.7)
DCR (95% CI)	76.2% (52.8–91.8)	0.0% (0.0–97.5)	0.0% (0.0–97.5)	0.0% (0.0–97.5)	100.0% (54.1–100.0)	80.0% (28.4–99.5)	100.0% (54.1–100.0)	100.0% (2.5–100.0)	80.0% (28.4–99.5)
DoR (months)									
Median (95% CI)	6.60 (3.77–13.3)	NE	NE	NE	NE	NE	13.3 (3.77–16.5)	6.60 (NE–NE)	5.58 (4.63–7.39)
PFS (months)									
Median (95% CI)	6.73 (4.73–8.54)	1.21 (NE–NE)	0.95 (NE–NE)	1.34 (NE–NE)	4.92 (NE–NE)	8.34 (3.02–26.9)	9.37 (4.69–17.7)	7.72 (NE–NE)	7.09 (4.73–8.54)
Follow-up time (months)									
Median (95% CI)	7.78 (4.82–9.69)	2.98 (NE–NE)	1.87 (NE–NE)	2.33 (NE–NE)	6.63 (NE–NE)	9.69 (4.13–NE)	9.90 (6.20–NE)	9.33 (NE–NE)	8.04 (2.82–NE)

*Abbreviations*: *ORR* objective response rate, *DCR* disease control rate, *PFS* progression-free survival, *DoR* duration of response, *CI* confidence interval, *NE* not evaluated, *QD* quaque die, *BID* bis in die

Among the 39 ALK TKI-naïve patients, 25 patients showed an objective response, the ORR was 64.1% (95% CI, 47.2–78.8) including 2.6% of CR and 61.5% of PR, and the DCR was 82.1% (95% CI, 66.5–92.5). The ORR in the dose-expansion cohort of 100mg QD (*n*=6), 450mg QD (*n*=8), 600 mg QD (*n*=12), and 300 mg BID (*n*=6) was 50.0% (95% CI, 11.8–88.2), 87.5% (95% CI, 47.3–99.7), 58.3% (95% CI, 27.7–84.8), and 50% (95% CI, 11.8–88.2), respectively, and the DCR were 66.7% (95% CI, 22.3–95.7), 100% (95% CI, 63.1–100.0), 75% (95% CI, 42.8–94.5), and 83.3% (95% CI, 35.9–99.6), respectively (Table 4).

For patients who received crizotinib previously, 7 of 21 patients achieved PR with an ORR of 33.3% (95% CI, 14.6–57.0), and 9 patients achieved SD with a DCR of 76.2% (95% CI, 52.8–91.8). The ORR in the dose-expansion cohort of 100mg QD (*n*=1), 450mg QD (*n*=5), 600 mg QD (*n*=6), and 300 mg BID (*n*=5) was 0% (95% CI, 0.0–97.5), 0% (95% CI, 0.0–52.2), 50% (95% CI, 11.8–88.2), and 60% (95% CI, 14.7–94.7), respectively, and DCR were 0% (95% CI, 0.0–97.5), 80% (95% CI, 28.4–99.5), 100% (95% CI, 54.1–100.0), and 80% (95% CI, 28.4–99.5), respectively (Table 4).

At the data cut-off date of February 28, 2022, for all patients (*n*=60), the median follow-up time was 9.75 months (95% CI, 6.63–12.5), median PFS was 9.26 months (95% CI, 6.73–15.7), and median DoR was 13.3 months (95% CI, 6.60–22.2) (Table 4).

In ALK TKI-naïve patients (*n*=39), the median follow-up time was 12.2 months (95% CI, 6.17–18.9), median PFS was 15.9 months (95% CI, 9.26–23.3), and median DoR was 15.0 months (95% CI, 9.06–25.8). Of note, within the dose cohorts that received conteltinib at 100 mg, 450 mg, 600 mg QD, and 300 mg BID, the median follow-up time was 12.7 months (95% CI, 3.74–NE), 17.1 months (95% CI, 2.75–52.5), 20.8 months (95% CI, 1.60–33.6), and 6.01 months (95% CI, 2.49–NE), respectively; median PFS was 11.3 months (95% CI, 2.75–15.9), NE (95% CI, 4.63–NE), 19.3 months (95% CI, 1.05–32.4), and 17.8 months (95% CI, 3.05–19.6), respectively; and median DoR was 11.1 months (95% CI, 5.61–15.0), NE (95% CI, 3.74–NE), 16.5 months (95% CI, 1.80–32.8), and 10.6 months (95% CI, 1.80–10.6), respectively (Table 4).

Of the patients who received crizotinib previously (*n*=21), the median follow-up time was 7.78 months (95% CI, 4.82–9.69), median PFS was 6.73 months (95% CI, 4.73–8.54), and median DoR was 6.60 months (95% CI, 3.77–13.3). Within the dose cohorts that received 100 mg, 450 mg, and 600 mg QD and 300 mg BID doses, the median follow-up time was 1.87 months (95% CI, NE–NE), 9.69 months (95% CI, 4.13–NE), 9.90 months (95% CI, 6.20–NE), and 8.04 months (95% CI, 2.82–NE), respectively; median PFS was 0.95 months (95% CI, NE–NE), 8.34 months (95% CI, 3.02–26.9), 9.37 months (95% CI, 4.69–17.7), and 7.09 months (95% CI, 4.73–8.54), respectively; and median DoR was NE, NE, 13.3 months (95% CI, 3.77–16.5) and 5.58 months (95% CI, 4.63–7.39), respectively (Table 4).

Notably, in the six patients with measurable brain metastatic lesions: 2 patients showed PR including 1 ALK TKI-naïve patient in 450 mg QD cohort and 1 ALK TKI-naïve patient in 600 mg QD cohort; 3 patients showed SD including 1 patient in 450 mg QD cohort who received crizotinib previously, 1 patient in 600 mg QD cohort who received crizotinib previously, and 1 ALK TKI-naïve patient in 300 mg BID cohort. In summary, conteltinib has achieved intracranial response with an ORR of 33.3% (2/6, 95% CI, 4.3–77.7) and DCR of 83.3% (5/6, 95% CI, 35.9–99.6) (Additional file 1: Table S2).

Considering the saturated drug absorption and potential high-grade toxicities, 800 mg QD was not selected for dose expansion nor as the recommended phase 2 dose (RP2D) for ALK TKI-naïve patients. Generally, 600 mg QD was identified to be the RP2D for ALK TKI-naïve patients owing to its better drug absorption, promising PFS, and manageable safety. Additionally, 300 mg BID was judged as the RP2D to achieve a better ORR and safety for ALK-positive patients who received crizotinib previously.

## Discussion

NSCLC is a cancer with high morbidity and mortality in the world. ALK-positive NSCLC accounts for about 3–5% in patients with NSCLC and is mostly diagnosed in the advanced stage [1, 2, 26]. ALK TKIs are still in an unmet clinical demand, which is largely due to their efficacy, safety concerns, and high price. It is urgent for multiple second-generation ALK TKIs to both improve therapeutic effects and safety profile, overcome relating resistances, and reduce the price as well.

Here, we present a first-in-human, phase 1 study in 64 patients and demonstrate that conteltinib (CT-707) has a manageable safety profile as well as favorable PK properties and anti-tumor activity, in patients with ALK-positive NSCLC. Based on these results, the RP2D were set as 600 mg QD of conteltinib for ALK TKI-naïve patients and 300 mg BID for patients who received crizotinib previously, respectively.

Conteltinib was well tolerated with a manageable safety profile. The MTD was not reached at 800 mg QD which was the highest dose cohort in this study. One DLT event was observed at the 600 mg once-daily dose cohort, which was reversed on discontinuation without affecting dose escalation. No treatment-related death was observed. Most grade 1–2 and grade ≥ 3 TRAEs were laboratory abnormalities that were resolved with standard supportive care. The safety profile of conteltinib was similar but not identical to other ALK TKIs. The incidence of grade ≥ 3 TRAEs was 14.1% in patients who received conteltinib in this study, while 24% in crizotinib [4], 49% in ceritinib [27], 36% in brigatinib [28], 23% in ensartinib [16], 26% in alectinib [29], and 35% in iruplinalkib [17]. In terms of drug-related gastrointestinal toxicities which are some of the most frequently reported AEs in ALK TKIs, diarrhea, nausea, and vomiting occurred in 35.9–71.9% patients in this study of conteltinib, mostly were grade 1–2 and in early stages of treatments, while 39–56% in crizotinib [4], 65–82% in ceritinib [27], 11–36% in ensartinib [16], 21–53% in brigatinib [28], < 10% in alectinib [29], and 14–34% in iruplinalkib [17]. Symptoms, such as visual effects and rash that were more frequently reported with crizotinib (64% visual effects, 11% rash) [4], ceritinib (13% rash) [27], ensartinib (56% rash; 12% grade ≥ 3) [16], and iruplinalkib (13% rash) [17], rarely occurred in this study of conteltinib (1.6% visual effects; 6.25% rash).

Conteltinib was well absorbed, with maximum concentrations in plasma at about 2 to 4 h and a single-dose half-life of roughly 20 to 90 h, which was similar to other ALK TKIs [16, 17, 27–30]. Over the dose range of 50 mg to 600 mg once daily, AUC and *C*_max_ increased in a manner beyond the dose proportion. However, the PK parameters did not show a significant change from 600 mg to 800mg (AUC_last_: 9644.2 vs. 9823.2 h ng/mL; *C*_max_: 452.1 vs. 421.3 ng/mL). As for the multiple-dose administration, the exposure of conteltinib was accumulated, continuously maintaining the effective anti-tumor concentration. Considering the saturated drug absorption and potential high-grade toxicities in dose > 600 mg QD, we did not expand the 800-mg QD dose cohort. As for the four selected doses in the dose-expansion phase, 600 mg QD demonstrated the best drug exposure and manageable safety profile in the dose-escalation phase, 450 mg QD was also suitable for expansion, 300 mg BID was suggested by PK data to maintain the efficacy and avoid potential toxicities by decreasing *C*_max_. Besides, preliminary efficacy was observed in the 100 mg QD cohort, and we started an expansion from this dose level according to the study protocol.

Conteltinib has effective anti-tumor activity not only in ALK TKI-naïve, advanced ALK-positive NSCLC patients (ORR=64.1%), but also in patients who received crizotinib previously (ORR=33.3%). For RP2D determination in ALK TKI-naïve, advanced ALK-positive NSCLC patients, an 800-mg QD dose of conteltinib was excluded owing to its saturated drug absorption and a high potential of high-grade toxicities. The 450 mg QD dose seems to show better clinical efficacy than the 600 mg QD dose; however, the relatively small sample size and large interpatient variability leave the dose to be further assessed. Generally, 600 mg QD yielded an ORR of 58.3% and a median PFS of 19.3 months and thus was assessed as the RP2D. For patients who received crizotinib previously, the 300 mg BID dose of conteltinib demonstrated a ORR of 60.0% and was better tolerated than the 600 mg QD dose and was determined to be the RP2D.

By integrating the published data, we indirectly compared the efficacy between conteltinib and other second-generation ALK TKIs. Overall, in ALK TKI-naïve patients, conteltinib received an ORR of 58.3% and median PFS of 19.3 months at RP2D, while an ORR of 59% and median PFS of 18.4 months were observed in ceritinib at MTD [27], an ORR of 80.0% and median PFS of 26.2 months in ensartinib at the dose around RP2D [16], an ORR of 93.5% and median PFS of 34.8 months in alectinib at the dose ≥ RP2D [29], an ORR of 100% and median PFS of 30.8 months in brigatinib at RP2D [19], and an ORR of 76.3% in iruplinalkib at the dose-expansion phase [17]. For patients who received crizotinib previously, conteltinib showed an ORR of 60.0% and median PFS of 7.09 months at RP2D, while an ORR of 56% and median PFS of 6.9 months were observed in ceritinib at MTD [13, 27], an ORR of 69.0% and median PFS of 9.0 months in ensartinib at the dose around RP2D [16], an ORR of 56.7% and median PFS of 9.6 months in alectinib at the dose ≥ RP2D [31], an ORR of 72% and median PFS of 12.9 months in brigatinib at RP2D [28], and an ORR of 45.7% and median PFS of 6.9 months in iruplinalkib at the dose-expansion phase [17]. Taken together, conteltinib exhibits comparable efficacy among the second-generation ALK TKIs.

Similar to most phase 1 studies, the limitation of this study was the small number of patients enrolled. To prospectively assess the efficacy and safety of conteltinib at RP2Ds, a multicenter, single-arm phase 2 study in ALK-positive, crizotinib-refractory NSCLC patients (CTR20181770) and a randomized phase 3 study of conteltinib versus crizotinib in patients with ALK TKI-naïve, advanced ALK-positive NSCLC (CTR20200770) are ongoing.

## Conclusions

In this study, conteltinib (CT-707) showed manageable safety profile, favorable PK properties, and anti-tumor activity in advanced ALK-positive NSCLC patients. The RP2D was determined to be 600 mg QD for ALK TKI-naïve patients and 300 mg BID for patients who received crizotinib previously.

## Supplementary Information

**Additional file 1: Table S1. ***In vitro* potency of crizotinib and conteltinib (CT-707). **Table S2.** Intracranial response of conteltinib (CT-707) in ALK-positive patients with brain metastasis.

## Data Availability

The datasets used and/or analyzed during the current study are available from the corresponding author upon reasonable request.

## Abbreviations

AE: Adverse event
AIDS: Acquired immunodeficiency syndrome
ALK: Anaplastic lymphoma kinase
ALT: Alanine transaminase
AST: Aspartate aminotransferase
AUC: Area under the concentration-time curve
AUC_INF_obs_: Area under the concentration-time curve from the time of dosing extrapolated to infinity, based on the last observed concentration
AUC_last_: Area under the concentration-time curve from the time of dosing to the time of the last observation
BID: Bis in die
*C*_max_: Maximum concentration
CR: Complete response
CT: Computed tomography
CTCAE: Common Terminology Criteria for Adverse Events
DCR: Disease control rate
DLT: Dose-limiting toxicity
DoR: Duration of response
ECOG: Eastern Cooperative Oncology Group
FISH: Fluorescence in situ hybridization
HIV: Human immunodeficiency virus
IHC: Immunohistochemistry
LVEF: Left ventricular ejection fraction
MRI: Magnetic resonance imaging
MRT_last_: Mean residence time from the time of dosing to the time of the last measurable concentration
MTD: Maximum tolerated dose
NE: Not evaluable
NSCLC: Non-small cell lung cancer
ORR: Objective response rate
PD: Progressive disease
PFS: Progression-free survival
PK: Pharmacokinetic
PR: Partial response
PS: Performance status
QD: Quaque die
RECIST: Response Evaluation Criteria in Solid Tumors
RP2D: Recommended phase 2 dose
RT-PCR: Reverse transcription-polymerase chain reaction
SAEs: Serious adverse events
SD: Stable disease
SS: Safety set
*T*_1/2_: Elimination half-life
TKI: Tyrosine kinase inhibitor
*T*_max_: Time to maximum concentration observed
TRAE: Treatment-related adverse event
ULN: Upper limit of normal
γ-GGT: γ-Glutamyl transpeptidase
