# Supplementary material for: Conteltinib (CT-707) in patients with advanced ALK-positive non-small cell lung cancer: a multicenter, open-label, first-in-human phase 1 study
Source: BMC Med. 2022 Nov 23;20:453. doi: 10.1186/s12916-022-02646-0 (PMC9694544; doi:10.1186/s12916-022-02646-0)
Supplement: Supplementary file 1 — Additional file 1: Table S1. In vitro potency of crizotinib and conteltinib (CT-707). Table S2. Intracranial response of conteltinib (CT-707) in ALK-positive patients with brain metastasis. [file 12916_2022_2646_MOESM1_ESM.docx]

| **Compound** | **ALK Enzymatic IC_50_ (nM)** | | | | | |
| --- | --- | --- | --- | --- | --- | --- |
|  | **WT** | **F1174L** | **L1196M** | **G1202R** | **G1269S** | **R1275Q** |
| **Crizotinib** | 4.0 | 6.7 | 62.5 | 30.4 | 211 | 6.1 |
| **Conteltinib** | 1.5 | 4.3 | 3.8 | 6.3 | 8.4 | 1.2 |

**Table S1. *In vitro* potency of crizotinib and conteltinib (CT-707)**

Abbreviation: ALK, anaplastic lymphoma kinase; WT, wild type; IC_50_, half-maximal inhibitory concentration.

**Table S2. Intracranial response of conteltinib (CT-707) in ALK-positive patients with brain metastasis**

| Intracranial Response | Total | 450 mg QD | 600 mg QD | 300 mg BID |
| --- | --- | --- | --- | --- |
| Patients (n) | 6 | 2 | 3 | 1 |
| ORR (95% CI) | 33.3% (4.3-77.7) | 50% (1.3-98.7) | 33.3% (0.8-90.6) | 0.0% (0.0-97.5) |
| DCR (95% CI) | 83.3% (35.9-99.6) | 100.0% (15.8-100.0) | 66.7% (9.4-99.2) | 100.0% (2.5-100.0) |

Abbreviation: ALK, anaplastic lymphoma kinase; ORR, objective response rate; DCR, disease control rate; CI, confidence interval; QD, quaque die; BID, bis in die.
